# Development and early experience from an intervention to facilitate teamwork between general practices and allied health providers: the Team-link study

**DOI:** 10.1186/1472-6963-10-104

**Published:** 2010-04-27

**Authors:** Mark F Harris, Bibiana C Chan, Christopher Daniel, Qing Wan, Nick Zwar, Gawaine Powell Davies

**Affiliations:** 1 Centre for Primary Health Care and Equity, University of New South Wales, UNSW, Sydney, 2052, Australia; 2 Central Sydney GP Network, 158 Liverpool Rd, Ashfield, 2131, Australia; 3 School of Public Health and Community Medicine, University of New South Wales, UNSW, Sydney, 2052, Australia

## Abstract

**Background:**

This paper describes the development and implementation of an intervention to facilitate teamwork between general practice and outside allied and community health services and providers.

**Methods:**

A review of organizational theory and a qualitative study of 9 practices was used to design an intervention which was applied in four Divisions of General Practice and 26 urban practices. Clinical record review and qualitative interviews with participants were used to determine the key lessons from its implementation.

**Results:**

Facilitating teamwork across organizational boundaries was very challenging. The quality of the relationship between professionals was of key importance. This was enabled by joint education and direct communication between providers. Practice nurses were key links between general practices and allied and community health services.

**Conclusions:**

Current arrangements for Team Care planning provide increased opportunities for access to allied health. However the current paper based system is insufficient to build relationships or effectively share roles as part of a patient care team. Facilitation is feasible but constrained by barriers to communication and trust.

## Background and Theory

Chronic disease management has become an increasing focus in the work of Australian general practice, as chronic conditions become more prevalent in the community and the expectation grows that they will be managed in primary health care [1]. Other countries are facing similar challenges in their health systems. Ongoing management of conditions such as diabetes, ischaemic heart disease and hypertension is complex and often requires multi-disciplinary care [2].

In Australia, most allied health services are provided outside general practice. The key models or approaches to chronic disease care in Australia include self management support, multidisciplinary care planning and coordinated care [1]. These models have been a major focus in primary care since the 1990s and in particular since multidisciplinary care plans were introduced as a part of the government-funded Medicare 1999 Enhanced Primary Care (EPC) package. They provided a mechanism for funding greater involvement by general practitioners (GPs), practice nurses and allied health professionals (AHPs) in structured and coordinated care. The "Team Care Arrangement" (TCA) was introduced to support multidisciplinary care for patients with chronic conditions and complex needs providing a rebate for up to five allied health services per year. This has considerably expanded the potential access for people with chronic diseases on low incomes to private AHPs and increased the involvement of AHPs in private practice in the care of patients with chronic disease.

Divisions of General Practice (DGPs) are Australia's main primary care organisations. On average there are about 200 General Practices (with over 300 GPs) in each Division. GPs participate in continuous professional development events run by DGPs which serve as a network for GP to share knowledge and expertise as well as facilitating integration of care with other providers. Each DGP provides many resources for GPs to access on-line as well as IT Support for their members. DGPs support practices through outreach visits which aim to facilitate improved quality care and the implementation of various initiatives including care planning.

Organising multi-disciplinary care in Australia is difficult, even when GPs are relatively well supported [3]. A qualitative study conducted in the initial phase of our project identified dissatisfaction with the referral and shared care processes on the part of general practice and allied health services [4], reflecting poor communication, limited exchange of information and difficulties in working relationships [5].

We therefore aimed to develop and evaluate an intervention (the Team-link study) that would enhance teamwork between general practice and public or private nursing and AHPs outside the practice for patients with diabetes or cardiovascular disease. This paper describes the development of the Team- link study and early lessons from its application. It follows the framework developed by the UK Medical Research Council for complex intervention research [6].

Theoretical frameworks of teamwork were sourced in the development of the intervention. We used case studies and descriptive research to inform the development and design of the intervention. We evaluated and reflected on the early lessons from the implementation to refine subsequent stages.

**Figure 1:**
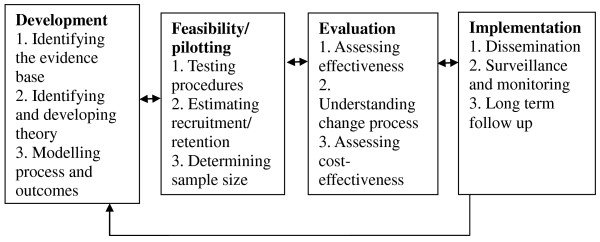
**Key elements of the development and evaluation process (adapted from **[6]).

Figure 1 summarises the four stages of the development of the Team-link study and the activities conducted at each stage (adapted from [6]).

## Theoretical basis of the intervention

Multidisciplinary care is characterized by a "team" responsible for the overall patient care plan [7]. Each individual team member contributes their own decisions and recommendations according to their specialty, which may be integrated by the team leader. Teamwork may also be considered as *'a dynamic process involving two or more healthcare professionals with complementary backgrounds and skills, sharing common health goals and exercising concerted, physical and mental effort in assessing, planning, or evaluating patient care'*. ([8], p. 232). Central to this approach is that the patient is involved in health care decisions as part of the team. In order to facilitate efficient and effective operation of a multidisciplinary team which is not co-located, careful planning and implementation is required.

The Team-link intervention was based on the theory of inter-professional care. In a review of the literature which dealt with concepts and definitions related to collaboration, D'Amour and colleagues found the main concepts related to the processes of collaboration were sharing, partnership, interdependency and power [9]. Simply bringing professionals together in teams does not guarantee collaboration; to be effective, multidisciplinary teams need clear objectives, roles and responsibilities as well as mechanisms for exchanging information and coordinating team activities [9].

Practitioners also need resources and tools to support teamwork and enable teams to achieve their objectives [10]. These include guidelines, structured protocols or policies and standards for communication. The size and composition of services and processes such as team meetings and auditing of current practice may also influence inter-professional team-working [11].

The College of Family Physicians of Canada [12] identified some key success factors for interdisciplinary collaboration in primary healthcare and the most important one being investing time in intra-group and inter-disciplinary communication. The core issues of 'Teamwork in General Practices' are summarised in Table 1 outlining the theories and intervention processes to address these issues.

**Table 1:** Core Issues of Teamwork in General Practices

Core Issues	Available Theoretical frameworks and Previous research	Intervention implemented
Engaging a multidisciplinary team with diverse expertise and approaches	**Tallia and colleagues **identified the importance of respecting each other's roles and acknowledging the diversity (of expertise) each member could bring to the team. Be mindful of new ways of working [14].	The GPs training workshop provided the initial information exchange between the multidisciplinary team. The facilitator's subsequent visits served as a bridge to link GPs to the AHPs with the appropriate skills and resources.

Trust (delegation)	**Stewart's Patient-centred Care Model **- GPs need to understand the roles played by AHPs and develop trusting relationships with them [15].	GPs Training workshops provide face-to-face interactions with AHPs where GPs and PNS were informed what kind of services AHPs offered and gauge the quality of such services during the case study discussions presented by the AHPs

Effective communication	**Tallia and colleagues **emphasised the importance of using rich means of communication (e.g. face-to face Vs faxed documents) [14]	A 3-way communication between GP-Patient-AHP via phone was modelled during the workshop and encouraged during follow up visits

Organizational support and team composition and location	**Xyrichis **pointed out the impact of team structure and processes on inter-professional team-working. E.g., team premises, size and composition, and availability of organizational support [11].	The facilitators provided the necessary resources and support for non-clinical staff (e.g. Practice Mangers or receptionists) of the practices, e.g. the appropriate software to update the computerised patient records and to run regular patient recalls, secure electronic transfer of patient data between the practice and AHPs, access to on-line resources available via the DGP's websites. The facilitators also helped staff members clarify their roles and responsibilities to avoid work duplications and promote efficiency.

Balance between focusing on tasks Vs social interactions	**The College of Family Physicians of Canada **identified 'investing time in intra-group' and 'inter-disciplinary communication' as key success factors for inter-disciplinary collaboration [12].	The facilitators encouraged the practice staff to have regular staff meetings to reflect on current issues related to chronic care management and exchange ideas, setting common goals and Plan-Do-Study-Act (PDSA) cycles. Regarding inter-disciplinary communication, the facilitators also organised 'Small Group Learning' Seminars at the local Division of General Practice for GPs and AHPs to exchange ideas on a particular topic of interest (e.g. Co-morbidities in patients with Chronic Disease)

Resources and Tools	**Sicotte **stated the need for resources and tools to support teamwork and enable teams to achieve their objectives [10].	The facilitators provided the necessary resources and tools to suit each practice's unique circumstances (IT support) as well as general tool-kits (e.g. TCA templates)

Bringing about changes in team processes require active facilitation; for example in a study of outreach facilitation of preventive care delivery in 30 primary care practices in Ontario [13], trained facilitators undertook practice visits to support changes in physician behavior, using strategies tailored to the context of individual practices.

Most previous research has been conducted within teams that are collocated or within the same organization. However this usually does not apply to patient care teams involving GPs and community and allied health services in Australia. Under these circumstances the relationship between team members is a critical pre-condition for effective collaborative work. Critical elements of these relationships include [14]:-

° Trust (delegation or referral of roles or tasks)

° Diversity of opinions, views and approaches within the team

° Mindfulness or openness to new ways of working

° Interrelatedness - understanding how each others' work effects one another

° Respect for each others' opinions and roles

° Varied interaction that is not simply task focused but allows social interaction

° Effective communication using rich means (eg face to face or phone, rather than faxed documents)

## Case studies

As part of developing the intervention, a series of case studies were conducted with nine practices of varying size in urban and rural areas of New South Wales, South Australia, Victoria and Queensland. In each practice GPs and practice staff (predominantly practice managers and practice nurses) were interviewed and transcripts analysed to generate a profile of how each practice operated as a team [16].

Most teamwork occurred within practices, with linkages to external services and providers being less common and quite limited in scope. These mostly involved AHPs contributing to care planning, but one small rural practice had meetings with community health staff. External linkages often required a designated link person within the practice. Few practices had protocols for referral or communication and most communication occurred via email or fax.

The interviews reflected the main issues found in the literature. Effective patient care teams required leadership, flexibility and a readiness to innovate. Informal communication such as interactions over lunch and more formal meetings were also required to ensure effective teamwork and to help build relationships. Roles and tasks needed to be clearly defined, especially in relation to care planning. Protocols and processes of care were often underpinned by the information systems within the practice, including recall systems, and this provided scope for expanding the role of the practice nurse. Training and education were important for enabling staff to take on new roles and to understand the roles of other providers and services.

## Design of the intervention

The Team-link study was designed to enhance communication and working relationships with service providers outside the general practice. Since this involved individual practices and allied health services from the area in which they were located, DGPs were an ideal vehicle to co-ordinate the intervention, and the facilitators were employees in the local DGPs with health-related backgrounds including: nursing, overseas medical training or public health. Facilitators were experienced in conducting in practice supported visits and were trained using an intervention workbook which described the aims of the study and the intervention, the processes to be undertaken in visits to each practice and the referral systems and resources available for each practice [see Additional file 1].

The intervention [see Additional file 2] began with a 2.5 hour evening workshop that brought GPs and practice nurses together with other nursing and AHPs involved in chronic disease care. This included AHPs from private and public sectors, including podiatrists, optometrists, diabetes educators, dietitians, cardiac rehabilitation workers, exercise physiologists and psychologists. At the workshop the intervention was described, principles of teamwork were discussed and a case study presented with a role played phone conversation for a prospective referral involving GP, patient and AHP.

This was followed over the next six months by three structured visits to each practice (each lasting 1-1.5 hours) where staff were introduced to the intervention resources. These included a practice work book, a referral directory, aids to referral (eg referral forms, cards with referral criteria), care plan templates, patient education materials (including Lifescripts resources: http://www.health.gov.au/internet/main/publishing.nsf/Content/health-pubhlth-strateg-lifescripts-genpracresources.htm) and billing systems for TCAs. Intervention activities were adapted to the particular priorities of each practice. Prior to each visit, staff were sent a meeting agenda and asked to familiarise themselves with Plan-Do-Study-Act (PDSA) cycles and a PDSA checklist for quality improvement. They were encouraged to identify areas for improvement and use the guidelines for system focused problem solving in the practice work book to formulate some small initial goals. The facilitator then assisted the practices to agree goals and monitor outcomes. Typical activities included using a speaker phone to involve patients in communication between the GP and the AHP, establishing information and referral systems, and setting up systems to ensure that the practice accessed the full range of Medicare incentives. Given the limits on GPs' time, these meetings usually lasted an hour.

Between structured visits, the facilitator provided ongoing support to the practices through informal visits or by phone. These involved troubleshooting problems and reviewing progress since the last visit, identifying what had and had not worked, and selecting new areas for improvement. This allowed the intervention to remain focused on the particular needs of the practice. The intervention facilitators also liaised with the allied health services by practice visits or telephone to facilitate referral and better support direct communication.

## Evaluation Framework

The intervention was implemented as part of a quasi-experimental study in four DGPs, with two Divisions receiving the intervention early and two later. The qualitative data were collected by the facilitators during the 6-month intervention delivered to 26 practices. These comprised three sources: 'Practice-visit reports' completed by the facilitators at the conclusion of each practice visit which summarized the content of the visit, the goals and strategies chosen by the practice and their progress in achieving them; evaluations reports (baseline and 6-months) completed by participating GPs which reflected on their experience in implementing the intervention, the barriers in multidisciplinary teamwork and possible strategies to overcome these barriers; and 'open-ended questions' completed by AHPs which addressed referral satisfaction, usefulness of TCAs and means of communication.

The 'Qualitative Analysis Framework' was based on D'Amour and colleagues model of the *concept of collaboration *[9]. This framework has been useful in establishing codes for theme analysis. The qualitative data were entered into NVivo 8 and coded thematically. There was cross coding by another author and this was then checked for discrepancies with differences resolved by discussion. This coding was used to extract the lessons from the implementation. The study was approved by the University of New South Wales Human Research Ethics Committee.

## Lessons from the implementation

While all GPs and practice nurses endorsed the need for better communication with other health service providers, some preferred to improve chronic disease care in the practice before addressing interactions with other service providers. Their PDSA cycles often focused first on establishing register and recall systems, patient education services or expanding activities (such as regular staff meetings) within the practice. However with encouragement and support most practices also focused on the TCAs and how they could improve the frequency of referral using directories of services.

Facilitators reported that for the PDSA cycles, small goals were more successful because they were more realistic in the six month timeframe of the study. Practices made considerable use of the resources and support from the division provided through the intervention, in particular IT support.

*Assisted the practice in using the Medical director software & TCA & new MBS item Templates. Discussed software updates with division's IT personnel. Agreed to provide ongoing assistance if necessary in the future." *(Intervention Facilitator, SW Sydney)

GPs reported changes in the way they managed patient care. Some recognised the need to collect more detailed histories from patients for developing care plans.

*Discussions with patients, regarding their care and the possible complications of their condition has taken place which has had a significant improvement on the patients turning up for their appointments." *(GP, Macarthur)

They reported having a better understanding of the roles of allied health service providers, and of the information that needed to be passed on to them. This flowed through into better patient satisfaction.

*The AHPs have firsthand knowledge of the patient's history prior to their appointment and therefore makes the patients treatment easier and faster. The patients are much happier with their treatment which makes them more compliant *(GP, Macarthur)

GPs noted that they felt more confident in referring patients to AHPs.

*Better understanding of Diabetes Clinic and services I am more confident in educating patients regarding the benefits of these services*. (GP attending small *group learning sessions, Central*)

Although the use of TCA templates was popular among GPs, these often did not suit the way AHPs worked with their patients or wished to communicate with GPs. While the emphasis in current care plans was on presenting most information in written form, both GPs and AHPs agreed that it was also important to get to know each other on a personal level to improve communication. While both groups recognised that time was short, they saw DGPs as well placed to organise joint 'Continuous Professional Development' events to strengthen professional relationships.

*Communication in both directions is vital to assist in not only developing an optimal system of referral, but a management partnership and possible learning environment" *(AHP in Macarthur).

The intervention highlighted the crucial role played by practice nurses in chronic disease management, with some GPs referring patients to practice nurses for follow up and patient education. Practice nurses indicated that such job delegation made it easier for them to work with patients. AHPs also welcomed the practice nurse's involvement as it improved communication.

[Before the study,] *communication within the practice was disjointed and now [the GP] is happy to delegate to others which reduces his workload*. (Intervention Facilitator, Macarthur)

*The practice Nurse's role speeds up any communication also*. (AHP Survey)

Some GPs considered building professional relationships and trust as a long term process which also required a strong commitment to teamwork.

*... there has not been enough passage of time to come to a conclusion as to whether there is better communications with allied health professionals at this time. It is an ongoing process*. (GP, Macarthur)

## Discussion and Conclusions

The components of the intervention in this study were based on the theory of inter-professional teamwork as applied to team members who were geographically dispersed across independent services and practitioners. The approach was adapted to the particular needs and priorities of the practices rather than attempting to impose a single approach. While this allowed practices to work on issues that were directly relevant to them, it also allowed them to focus on internal systems and teamwork within the practice because it was more directly within their influence.

Assisting practices to reach out to external AHPs was more challenging. Although the intervention facilitators made contact (mainly by phone) with some of the AHPs involved with the participating practices and some attended the evening workshops with GPs and practice nurses, there was no structured intervention for them. Future research should involve AHPs and assist them to improve their relationship and communication with GPs as well as vice versa.

The observations by facilitators in this study confirmed the importance of mutual understanding of each other's roles, of developing professional relationships and enhancing communication to improve inter-professional working. It was clear that although the TCAs are designed to facilitate access to allied health, they are not effective in encouraging the two way communication or the trust that is essential for team care. This requires more active facilitation (Table 2). The lessons learnt from this study provide an important basis for facilitating teamwork through DGPs and for the development of new models of primary health care envisaged by the health reform process (see A *Healthier Future For All Australians - Final Report *for more details: http://www.health.gov.au/internet/nhhrc/publishing.nsf/Content/nhhrc-report).

Our Team-link experience also suggests that facilitation is feasible but constrained by structural barriers to trust and communication (such as distance and funding based on encounters with individual providers). Our intervention facilitators, who used a tailored approach in each practice, were also constrained by priorities of the general practice staff which tended to be inwardly focused. Communication and teamwork outside of the practice were a lower priority for many primary care staff than establishing better team care systems within the practice.

There are many competing interests for the GPs in terms of running a sustainable (financially) medical practice and providing the best clinical care to their patients. The interviews with GPs suggested that there seemed to be little incentive (both tangible and non-tangible) to change the 'culture' from working independently as a primary health care service provider to part of a team in multidisciplinary care in chronic disease management. The Australian Government initiatives in introducing Medicare items such as the TCAs to pay (or compensate) for the time GPs use to coordinate 'Care Planning' provided only a modest financial incentive, when balanced against the 'paper work'[4].

## Limitations of the study

There are a number of limitations to this study which need to be considered in interpreting the results. The evaluation was based on written observations and the self-reported experiences of facilitators, general practitioners and AHPs. The time-frame of the intervention was relatively short to achieve improvements in patient outcomes. The intervention was predominantly general practice based whereas future interventions should consider engaging AHPs more directly in the intervention process. A longer time-frame may provide more evidence of improved patient outcomes.

While incentives for collaborative care have been introduced in recent years, these do not nearly create effective team care. This requires active facilitation. The team-link study has demonstrated that GPs and AHPs can engage in multidisciplinary care of patients with minimal facilitation. This is an area that has received comparatively little research attention despite its importance.

**Table 2:** Extract from an intervention facilitator's practice visit reports, modified to protect anonymity

	Needs	**1**^**st**^** Visit**	**2**^**nd **^**Visit**	**3**^**rd**^** Visit**
**Staff Roles**	Role specification and clarification Dedicated person to contact the AHPs	Clarify the different roles of practice staff - practice manager (PM), nurse (PN) and receptionist. Regarding referral: practice manager would be the key person available for other health professions to contact.	Practice has currently established the guidelines of Team Link project. (The facilitator) Encouraged the practice to organizing a staff meeting in view of strengthening the internal team.	Introduced new strategies to deal with the ever increasing amount of paperwork. Regular staff meetings required to discuss ways of improving chronic disease management and how to optimize the existing resources.

**Communication**	Foster better interpersonal relationship with AHPs Investigate the quality and availability of services by AHPs in private practice Contact details required frequent updates	Consider face to face meeting with AHPs	An improvement in communication with AHPs noticed, however the feedback from the AHPs regarding the referrals is expected to improve with time. The practice was visited & followed up by several AHPs frequently on demand. Exercise Physiologist, Podiatrist, Psychologist & Dentist are among them. Practice is currently seeking the service of a Dietitian. The Division is trying to refer them to a reliable Dietitian who can visit the practice when necessary. PM has an idea of allocating a staff member (PN) to update & maintain their AHPs database.	Significant improvement observed in the feed back process from AHPs as result of communicating via email and the feedback is electronically archived for each patient in the database. They are finalizing arrangements to recruit the dietitian. Organized a dietitian, he is paying regular visits to the practice. A staff member has been allocated to update and change database.

**Systems for Recall**	Set up effective operational recall system	Arrange staff to improve patient recall system Practice manager will update the new Medicare items, eg 75 health check	They have improved the CDM registers & recall systems. They are currently in the process of updating Medical Director Software & its data bases and seek some assistance from our IT department.	

**Patient Education**	Need for patient to self-manage for patient safety and patient empowerment		The ongoing plan in management of patients with chronic diseases is constantly reviewed & more patient education sessions have to be organized. They ask for some resources (Educational materials/pamphlets etc) from us.	Supplied with educational materials (i.e. pamphlets leaflets etc.) for patients on general self management. Initiating a patient education session

## Conflict of interests

The authors declare that they have no competing interests.

## Authors' contributions

MH, NZ and GPD conceived of the study, participated in its design and coordination, and helped to draft the manuscript. BC coordinated the implementation of the study and drafted the manuscript. CD and QW implemented the intervention and drafted the manuscript. All authors read and approved the final manuscript.

## Pre-publication history

The pre-publication history for this paper can be accessed here:

http://www.biomedcentral.com/1472-6963/10/104/prepub

## Supplementary Material

Additional file 1 **Appendices - Resources for the Team-link Project**. This document contains all the resources provided to the practices participating in the team-link project. Click here for file

Additional file 2 **A snapshot of the 'Team-link' project**. This document provides a snapshot of the 'Team-link' project protocol. Click here for file
