# Supplementary material for: Development and early experience from an intervention to facilitate teamwork between general practices and allied health providers: the Team-link study
Source: BMC Health Serv Res. 2010 Apr 27;10:104. doi: 10.1186/1472-6963-10-104 (PMC2877025; doi:10.1186/1472-6963-10-104)
Supplement: Additional file 1 — Appendices - Resources for the Team-link Project. This document contains all the resources provided to the practices participating in the team-link project. [file 1472-6963-10-104-S1.DOC]

**Additional Files**

**Additional File 1**

File Format: DOC

Title: Appendices – Resources for the Team-link Project

Description: This document contains all the resources provided to the practices participating in the team-link project.

Referral information: A prompt sheet

, o

Reason for referral:

Diagnosis

Assessment/ management

What specific goals are to be met from this referral?

Format:

Typed letters

Structured

Brief. If a lot of information needs to be included, a summary may come in handy

Relevant information:

- Previous related investigations/ treatments and the outcomes of these
- Patient’s current management plan/treatment
- Follow up arrangements (e.g. how often review, reviewed by whom)
- What the patient has been told about his/her condition and about what to expect from the referral/referral service

Background information:

- Psychosocial situation that may impact on management (e.g. motivation/compliance, depression, access issues).
- Family history, etc.
- Language or cultural issues that may affect management

CHECK:

- If you are able to, contact the other health professional by phone, fax, or email prior to referral. This is particularly the case if putting the patient on a Team Care Arrangement.
- Patient consent for release of information
- Who to send copies to (e.g. diabetes educator, dietician)

Roles and responsibilities of health professionals involved in chronic disease care

Diabetes educator

| What they do | - Educate patients about diabetes and how to manage it, emphasising the importance of self-management. - Focus on helping the patients understand diabetes and the importance of medication, blood glucose monitoring, lifestyle factors (diet, exercise), and foot care. - Help patients develop an individualised diabetes self-management plan. - Specialise in diabetes so they keep up to date with the latest information. |
| --- | --- |
| Training/ qualifications | - Often are university-trained nurses or dieticians who have undertaken further training specialising in diabetes education and management. - Professional body – Australian Diabetes Educators Association (ADEA). |
| Best time to link in | - Early on following diagnosis. - After that, as required as ‘booster’ sessions. |

Podiatrist

| What they do | - Assess and treat patients’ feet for signs of nerve damage and circulation impairment related to diabetes. - Educate patients about the link between high blood glucose levels and persistent foot problems. - Educate patients regarding the self-management of foot care to prevent serious problems. |
| --- | --- |
| Training/ qualifications | - University-trained in podiatry. - Professional body – Australasian Podiatry Council. |
| Best time to link in | - Early on following diagnosis. - After that, at least every 6 months for patients with diabetes. - Seek care for persistent infections or sores that are slow to heal. |

Dietician

| What they do | - Educate patients in detail about the impact of food on chronic diseases such as diabetes and ischaemic heart disease. - For patients with diabetes, dietitians can educate patients about the food they eat affects their blood glucose levels. - For patients with ischaemic heart disease, dietitians can educate patients about healthy food choices for a healthy heart. - Help patients to develop healthy eating plans. - Provide up-to-date knowledge in dietetics and nutrition. |
| --- | --- |
| Training/ qualifications | - University-trained in nutrition and dietetics. - Professional body – Dieititians’ Association of Australia. |
| Best time to link in | - Early on following diagnosis. - After that, as required as ‘booster’ sessions. |

Endocrinologist

| What they do | - Provide specialist medical services for patients with complicated problems related to diabetes. - Work together with GPs and other professionals to help patients control their blood glucose levels. |
| --- | --- |
| Training/qualifications | - University trained in medicine plus additional specialist medical training (entailing over 8 years of training). - Professional body – Australian Medical Association, Royal Australasian College of Physicians. |
| Best time to link in | - Patients who experience complications in diabetes management and whose diabetes is unstable. - Early on for stabilising |

Ophthalmologist/Optometrist

| What they do | - Monitor patients with diabetes for retinopathy and maculopathy. This can be done by both the optometrist and the ophthalmologist. - Treatment of diabetic retinopathy and maculopathy (ophthalmologist). |
| --- | --- |
| Training/qualifications | - Bachelor degree or higher (optometrist); bachelor degree or higher plus at least 5 years specialist training (ophthalmologist) - Professional body – Australian Medical Association, Royal Australasian College of Physicians, Optometrists Association Australia |
| Best time to link in | - Following diagnosis, with regular monitoring at least once every 2 years. |

Psychologist/social worker/mental health worker

| What they do | - Address psychosocial issues that affect the patient’s ability to engage in the self-management of diabetes/ischaemic heart disease. These may include lifestyle change, stress, depression, smoking, weight management, and motivation. |
| --- | --- |
| Training/qualifications | - University trained (psychologist, social worker, psychiatrist) plus additional specialist training (psychologist, psychiatrist). - Professional bodies – Australian Psychological Society, Australian Medical Association, Royal Australian and New Zealand College of Psychiatrists, Australian Association of Social Workers. |
| Best time to link in | - When patients experience difficulties with self-management due to psychosocial factors. |

Dentist

| What they do | - Examine patients with diabetes for dental and periodontal problems. |
| --- | --- |
| Training/qualifications | - University trained - Professional body – Australian Dental Association |
| Best time to link in | - At least once a year |

Cardiac rehabilitation worker

| What they do | - Assist patients to return to an active and satisfying lifestyle by targeting the following areas – exercise, education, and psychological and social support. - Educate patients about lifestyle factors in maintaining a healthy heart; help to develop a self-management program. |
| --- | --- |
| Training/qualifications | - Cardiac rehabilitation workers are multidisciplinary professionals with an interest in cardiac care. Professions involved in cardiac rehabilitation include nurses, exercise physiologists/physiotherapists, social workers, psychologists, pharmacists, dietitians, and Aboriginal health workers. |
| Best time to link in | - As an inpatient /outpatient, with maintenance sessions as needed. |

Cardiac failure liaison nurse

| What they do | - Follow up patients with heart failure after hospital discharge to provide support and education and try to reduce rates of re-admission. - Liaise with carers in the community such as GPs to improve care in the community. |
| --- | --- |
| Training/qualifications | - Nurses with experience in working in cardiology or the community. |
| Best time to link in | - Following a hospital admission for a heart failure patient. |

Pharmacist

| What they do | - The pharmacist plays an important role in monitoring the combination of medications that patients with chronic diseases may be on. |
| --- | --- |
| Training/qualifications | - University trained - Professional body – Pharmaceutical society of Australia, |
| Best time to link in | - When patient commences new medication, the pharmacist can review medications for interactions. - Regular contact to review medications. |

Cardiologist

| What they do | - Provide specialist medical care for heart disease. |
| --- | --- |
| Training/qualifications | - University trained plus additional specialist medical training. - Professional body – Australian Medical Association, Royal Australasian College of Physicians. |
| Best time to link in | - When patients experience complications in the management of ischaemic heart disease. |

Aboriginal health worker

| What they do | - Provide culturally appropriate and holistic care for Indigenous patients with diabetes/ ischaemic heart disease. - May do basic clinical measurements (pulse, blood pressure, respirations, and blood sugar levels) or more advance clinical skills depending on level and type of qualification undertaken. - Cultural health assessments and culturally appropriate health education, incorporating cultural, social and emotional and spiritual wellbeing. - Community development activities, primary health care and health promotion programming. |
| --- | --- |
| Training/qualifications | - University trained: Undergraduate qualification in Aboriginal Health, Primary Health Care or Health science. |
| Best time to link in | - Following diagnosis for education and self-management - Ongoing care for regular check-up and monitoring. |

Exercise physiologist

| What they do | - Conduct fitness assessments and develop an exercise management plan - Education regarding the role of exercise in managing chronic diseases |
| --- | --- |
| Training/qualifications | - University trained - Professional body – Australian Association for Exercise and Sports Science. |
| Best time to link in | - Following diagnosis for education and development of management plan. - Ongoing care for regular check-up and monitoring. |

| **Chronic Disease Management (CDM) - Checklist for GPs**  **Coordinating Team Care Arrangements (TCA) – MBS Item 723** |
| --- |

- GPs coordinating a TCA should refer to Medicare item 723 and the relevant Medicare Benefits Schedule (MBS) explanatory notes before using this checklist – see www.health.gov.au/chronicdisease.
- Use of this checklist is not mandatory.
- Checklists for all CDM items are available at the above website.

| **1. Eligibility** | | |
| --- | --- | --- |
| This service is available to patients in the community and to private in-patients (including residents of aged care facilities) being discharged from hospital (see Medicare Item Note A.22.22).  This service is not available to public in-patients being discharged from hospital or residents living in an aged care facility.  This service is for patients with a chronic or terminal medical condition and who require ongoing care from a multidisciplinary team. [See Medicare Item Note A.22.15].  Patients with a TCA (item 723) and a GP Management Plan (GPMP - item 721) are eligible for rebates under the allied health and dental care items (nos. 10950 to 10977) – see Medicare Item note A.22.20 for details. | | |
| **2. Pre TCA** | | |
| Would the patient benefit by having a TCA? |  | Mandatory |
| Explain the steps and any costs involved in a TCA to the patient |  | Mandatory |
| Record the patient’s agreement to proceed |  | Mandatory |
| Obtain relevant information (eg GPMP, previous care plans) |  | Recommended |
| **3. Team Care Arrangements (TCA)** | | |
| **This includes the steps as per Note A22.17 in the MBS:** | | |
| - Discuss with the patient which treatment/service providers should be asked to collaborate with the GP in completing TCA |  | Mandatory |
| - Gain the patient’s agreement to share relevant information |  | Mandatory |
| - Contact the proposed providers and obtain their agreement to participate |  | Mandatory |
| - Collaborate with the participating providers to discuss potential treatment/services to achieve management goals for the patient |  | Mandatory |
| - Document the goals, the collaborating providers, the treatment/services they have agreed to provide, patient actions and a review date i.e. complete the TCA document (may be documented as an addition to the patient’s GPMP) |  | Mandatory |
| Offer a copy of the TCA to the patient (and their carer if the patient consents) |  | Mandatory |
| Provide relevant parts/a copy of the TCA to the other providers in the team |  | Mandatory |
| Copy of the TCA added to patient’s medical record |  | Mandatory |
| With patient’s agreement, provide copy of TCA or relevant parts to other providers involved in the patient’s care. |  | As appropriate |
| Use an *EPC Program referral form for allied health services under Medicare* when referring patients to allied health professionals. |  | Mandatory (if referring) |
| **4. Ongoing Management and Review** | | |
| Manage the patient’s needs through normal consultations and regular review, using TCA Review (MBS Item 727) or GPMP Review (MBS Item 725) as appropriate |  | As indicated |

Reproduced with the permission of Central Sydney Division of General Practice, available at <http://csdgp.com.au/downloads.htm>

| **CHRONIC DISEASE MANAGEMENT**  **TEAM CARE ARRANGEMENTS (MBS ITEM No. 723)** |
| --- |

**SAMPLE FORM No. 723**

| **Patient’s Name:** ………………………… | **Date of Birth:** ………………………… |
| --- | --- |
| **Contact Details:**  …………………………………………  …………………………………………  ………………………………………… | **Medicare or Private Health Insurance Details:**  ………………………………………  ………………………………………  ………………….………………… |
| **Details of Patient’s Usual GP:**  ………………………………………………  ………………………………………………  ……………………………………………… | **Details of Patient’s Carer (if applicable):**  ………………………………………………  ………………………………………………  …………………………………………… |
| **If the patient has a previous or existing care plan, when was it prepared and what were the outcomes?**  ……………………………………………………………………………………………………..  ……………………………………………………………………………………………………………………………………………………………………………………………………………………………………………………………………………………………………………………… | |
| **Other notes or comments relevant to the patient’s care planning**  ……………………………………………………………………………………………………  ……………………………………………………………………………………………………  ……………………………………………………………………………………………………  ……………………………………………………………………………………………………  ……………………………………………………………………………………………………  ……………………………………………………………………………………………………  …………………………………………………………………………………………………… | |
| **MEDICATIONS**  ……………………………………………………………………………………………………  ……………………………………………………………………………………………………  ……………………………………………………………………………………………………  …………………………………………………………………………………………………… | |
| **ALLERGIES**  ……………………………………………………………………………………………………  ……………………………………………………………………………………………………  …………………………………………………………………………………………………… | |

Reproduced with the permission of Central Sydney Division of General Practice, available at <http://csdgp.com.au/downloads.htm>

**Patient’s name: …………………………………………………………………………………………………………………………………………………………………………..**

| **I have explained the steps and costs involved and the patient has agreed to proceed with the service. The patient also agrees to the involvement of other health providers and to share their clinical information without/with restrictions (identify)…………………………………………………………………** (GP’s Signature & Date) |
| --- |

| **TEAM CARE ARRANGEMENTS** | | | | |
| --- | --- | --- | --- | --- |
| Goals – Changes to be achieved. | Requiring treatments and services including patient actions | | | Arrangements for treatment/services (when, who, contact details) |
|  |  | | |  |
| **Copy of TCA offered to patients?** YES / NO | | **Copy/relevant parts of the TCA supplied to other providers?** YES / NO | | |
| **TCA added to the patient’s records?** YES / NO | | **Referral forms for Medicare allied health and dental care services completed?** YES/NO  [For referral forms call 1800 067 307 or go to www.hic.gov.au/providers/forms] | | |
|  |  | | |  |
| **Date service was completed:** …………………………………………………………. | | | **Review Date:** …………………………………………………………………………………….. | |

Reproduced with the permission of Central Sydney Division of General Practice, available at <http://csdgp.com.au/downloads.htm>

| Chronic Disease Management - Checklist for GPs  **Preparing a GP Management Plan (GPMP) – MBS Item 721** |
| --- |

- GPs undertaking a GPMP should refer to Medicare item 721 and the relevant MBS explanatory notes before using this checklist – see www.health.gov.au/chronicdisease.
- Use of this checklist is not mandatory.
- Checklists for all CDM items are available at the above website.

| **1. Eligibility** | | |
| --- | --- | --- |
| This service is available to patients in the community and to private in-patients (including residents of aged care facilities) being discharged from hospital (see Medicare Item Note A.22.13).  This service is for patients with a chronic or terminal medical. [See Medicare Item Note A.22.42(a)].  This service is not available to public in-patients being discharged from hospital or residents living in an aged care facility. | | |
| **2. Pre GPMP** | | |
| Would the patient benefit by having a GPMP? |  | Mandatory |
| Explain the steps and any costs involved to the patient |  | Mandatory |
| Record the patient’s agreement to proceed |  | Mandatory |
| Obtain relevant information (eg previous care plans or assessments) |  | Recommended |
| **3. GP Management Plan** | | |
| **This includes the steps as per Note A22.12 in the MBS:** | | |
| - Assess the patient – identify &/or confirm health care needs, problems and relevant conditions |  | Mandatory |
| - Agree on management goals with the patient (changes to be achieved by the treatment and services identified in the plan) |  | Mandatory |
| - Identify any actions to be taken by the patient |  | Mandatory |
| - Identify required treatment and services; make arrangements for provision of these services and for ongoing management |  | Mandatory |
| - Document the patient’s needs, goals, patient actions, treatment/services and a review date (i.e. completing the GPMP document) |  | Mandatory |
| Offer a copy of the GPMP to the patient (and their carer if the patient consents) |  | Mandatory |
| Copy of the GPMP added to patient’s medical record |  | Mandatory |
| With patient’s agreement, provide copy of GPMP or relevant parts to other providers involved in the patient’s care. |  | As appropriate |
| **4. Ongoing Management and Review** | | |
| Manage the patient’s needs through normal consultations and regular review, using GPMP Review (MBS Item 725) or TCA Review (MBS Item 727) items as appropriate |  | As indicated |

Reproduced with the permission of Central Sydney Division of General Practice, available at <http://csdgp.com.au/downloads.htm>

| **CHRONIC DISEASE MANAGEMENT**  **GP MANAGEMENT PLAN (MBS ITEM No. 721)** |
| --- |

**SAMPLE FORM No. 721**

| **Patient’s Name:** ………………………… | **Date of Birth:** ………………………… |
| --- | --- |
| **Contact Details:**  …………………………………………  …………………………………………  ………………………………………… | **Medicare or Private Health Insurance Details:**  ………………………………………  ………………………………………  ………………….………………… |
| **Details of Patient’s Usual GP:**  ………………………………………………  ………………………………………………  ……………………………………………… | **Details of Patient’s Carer (if applicable):**  ………………………………………………  ………………………………………………  …………………………………………… |
| **If the patient has a previous or existing care plan, when was it prepared and what were the outcomes?**  ……………………………………………………………………………………………………..  ……………………………………………………………………………………………………………………………………………………………………………………………………………………………………………………………………………………………………………………… | |
| **Other notes or comments relevant to the patient’s care planning**  ……………………………………………………………………………………………………  ……………………………………………………………………………………………………  ……………………………………………………………………………………………………  ……………………………………………………………………………………………………  ……………………………………………………………………………………………………  ……………………………………………………………………………………………………  …………………………………………………………………………………………………… | |
| **MEDICATIONS**  ……………………………………………………………………………………………………  ……………………………………………………………………………………………………  ……………………………………………………………………………………………………  …………………………………………………………………………………………………… | |
| **ALLERGIES**  ……………………………………………………………………………………………………  ……………………………………………………………………………………………………  …………………………………………………………………………………………………… | |

Reproduced with the permission of Central Sydney Division of General Practice, available at <http://csdgp.com.au/downloads.htm>

**Patient’s name: …………………………………………………………………………………………………………………………………………………………………………..**

| **I have explained the steps and costs involved, and the patient has agreed to proceed with the service. …………………………………………** (GP’s Signature & Date) |
| --- |

| **GP MANAGEMENT PLAN** | | | | | | |
| --- | --- | --- | --- | --- | --- | --- |
| Patient problems / needs / relevant conditions | Goals – changes to be achieved | | | Required treatments and services including patient actions | | Arrangements for treatments/ services (when, who, contact details) |
|  |  | | |  | |  |
| **Copy of GPMP offered to patients?** YES / NO | | | **Copy/relevant parts of the GPMP supplied to other providers?** YES / NO/ NOT REQUIRED | | | |
| **GPMP added to the patient’s records?** YES / NO | | |  | | | |
|  | |  | | |  | |
| **Date service was completed:** ………………………………………………………… | | | | **Review Date:** …………………………………………………………………………………….. | | |

Reproduced with the permission of Central Sydney Division of General Practice, available at <http://csdgp.com.au/downloads.htm>

Plan-Do-Study-Act

What is the goal?

PDSA 1

| **Goal:** | |
| --- | --- |
| **P** |  |
| **D** |  |
| **S** |  |
| **A** |  |

PDSA 2

| **Goal:** | |
| --- | --- |
| **P** |  |
| **D** |  |
| **S** |  |
| **A** |  |

PDSA evaluation sheet

**GOAL:**

What did you do to achieve this goal (i.e. outline goal in detail)?

__________________________________________________________________________________________________________________________________________________________________________________________________________________________________________________________________________________________________________________________________________________________________________________________________________________

What worked?

What didn’t work?

What could you have done differently?

What other areas for improvement has this PDSA cycle exposed?

Using your PDSA evaluation sheet

Step 1: Write down the goal.

Step 2: List what you did (in detail) to achieve this goal.

Step 3: Reflect on the following:

*What worked?*

What worked, and why did it work? The answer to these questions will cue you to successes within your practice, and will also uncover ways of working within your practice that are easy adopted by practice staff. For example, if your PDSA cycle was successful in increasing communication with other services surrounding shared patients, look at what it was about this PDSA cycle that made it successful. Did certain staff members find it easier to implement this cycle than others? If so, they may be natural communicators who will be an asset in a liaison role. Did some find it easier to establish a system for communication with other services? If so, they may excel in roles where there is great attention to detail. Knowing why and how something worked makes it easier for your practice to plan future PDSA cycles to maximize staff interests and abilities.

*What didn’t work?*

This will be the opposite of what worked, and will cue your practice to ways of doing things that are not efficient in your practice. For example, if achieving a goal required the input of several staff members, with the turnaround from each staff member associated with a short delay, then this signals that a different way of working may be required. Perhaps this means breaking down the larger goal into smaller goals with different ‘teams’ of a few staff members involved in completing any one goal.

*What could you have done differently?*

Examining what you could have done differently in carrying out the PDSA cycle encourages the practice staff to think creatively about approaching the issue from different perspectives. Brainstorming amongst practice staff may help encourage new ways of working.

*Areas for further improvement that this PDSA cycle exposed*

New areas for improvement may be exposed because a PDSA cycle was successful (for example, the same techniques may be generalized to other areas), or because it was unsuccessful (for example, the PDSA cycle did not work because a critical part was not already in place within the practice). Irrespective of the level of success, use the outcome of your PDSA cycle to help inform your practice on different areas of working.
