# Supplementary material for: Development and early experience from an intervention to facilitate teamwork between general practices and allied health providers: the Team-link study
Source: BMC Health Serv Res. 2010 Apr 27;10:104. doi: 10.1186/1472-6963-10-104 (PMC2877025; doi:10.1186/1472-6963-10-104)
Supplement: Additional file 2 — A snapshot of the 'Team-link' project. This document provides a snapshot of the 'Team-link' project protocol. [file 1472-6963-10-104-S2.DOC]

**Additional File 2**

**File format: DOC**

**Title: A snapshot of the ‘Team-link’ project**

***‘Team-link at a glance’***

The ‘Outcomes of Multidisciplinary Care in General Practice’ (or the Team-link) Project was funded by the Australian Health Ministers Advisory Council (AHMAC) from July 2006 – June 2010. This project aimed at investigating the impact of an intervention to enhance working relationships between general practice and other services in the shared care of patients with chronic diseases. Diseases of focus weree Type 2 diabetes, ischaemic heart disease, or hypertension. There was a secondary focus on enhancing teamwork within general practice to facilitate the shared care processes mainly on the issues of practice capacity and communication.

This study adopted a quasi-experimental, cluster delayed intervention design. The duration of this study is 12 months. Two Divisions of General Practice DGPs and their participating practices) were allocated to the early and two DGPs delayed intervention condition. Allocation was determined so as to minimise contamination – some referral services are shared between different DGPs, thus for instance these Divisions will be allocated to both receive, say, the delayed intervention.

The intervention is a 6 month program:

0 mths

6 mths

12 mths

Early

Delayed

Participating practices received a 6 month program to 1) enhance referral relationships with referral services, and 2) enhance teamworking within the practice to support sharing the care of patients with Type 2 Diabetes and/or ischaemic heart disease/hypertension.

This program evaluated according to 1) patient health outcomes (both objective and self-report); 2) quality of care; and 3) the process of sharing care/referral relationships.

Practice evaluation measures were administered on two occasions – pre- and post-intervention (0 and 6 months for Early, 6 and 12 months for Delayed). Patient evaluation measures were administered on three occasions (except for clinical audit which will be conducted once) to correspond with an annual cycle of care (0, 6, and 12 months).

***Divisions***

- Four Divisions of General Practice were recruited to the study; 2 in the early intervention condition and 2 in the delayed intervention condition.
- The study was conducted in partnership with the following DGPs:
  - Central Sydney Division of General Practice
  - South Eastern Sydney Division of General Practice
  - Macarthur Division of General Practice
  - Fairfield and Liverpool Division of General Practice
    - Each Divisions signed a Memorandum of Understanding with UNSW, which outlines roles, responsibilities, and commitments of DGPs and UNSW,

***Practices***

- Altogether, 26 practices were recruited.
- Criteria for inclusion in the study are:
  - Use of computer prescribing
  - Have two or more people working in the practice
  - Have a non-GP practice manager, practice nurse, or receptionist role
- The project provided
  - Tailored support for 6 months from Division-based project officer who assisted practice with setting small-scale projects (PDSA cycles) to enhance working relationships with other services. Focus on areas including:
    - Making teamwork financially viable
    - Improving communication with other services
    - Enhancing referral and reply information exchanged in the shared care of patients
    - Establishing processes so that sharing care is more effective and more efficient
  - Participation in data collection to examine patient health outcomes, quality of care, and working relationships. Includes questionnaires, interviews, audit of eligible patients’ records, and practice claims for relevant Medicare items (e.g. TCAs).

***Referral services***

- The number of referral services ‘recruited’ to the study will depend on the existing referral relationships held with general practices. Aim to include:
  - Public services (e.g. cardiac rehabilitation clinic, diabetes clinics)
  - Some private referral services
  - Some medical specialists (endocrinologists, cardiologists)

***Patients***

- A total of 1600 patients (40 patients per practice) will be recruited to the study.
- Practices will be assisted by UNSW Evaluation Officer to identify eligible patients.
- Patient eligibility criteria:
  - Over 18 years old
  - Have Type 2 Diabetes, ischaemic heart disease, and/or hypertension
  - Have been at the practice for the past 18 months

***Measures***

- - Measures will be collected at the patient, practice, practitioner, and referral service levels. These relate to the quality of care, health status of patients, job satisfaction and referral relationships.

*Practice measures*

- - Practice claims of HIC payment for Team Care Arrangements, completed cycles of care, and GP Management Plan.
  - Team climate inventory (all staff to complete)
  - Clinical Care Inventory (GP)
  - Practice Profile Inventory (Condensed based on factor analysis, plus linkages section; GP or PM to complete)
  - Measure of Multidisciplinary Linkages (GP version – to examine working relationships)
  - Referral content (audit of letters)
  - Pre-Practice Visit Questionnaire (GP or PM to complete)

*Referral service measures*

- - Measure of Multidisciplinary Linkages (Referral Service version)

*Patient measures*

- - Audit of medical records to determine
    - Patient health status (dates and values of blood pressure, HbA1c, cholesterol, weight, blood pressure),
    - Multidisciplinary care (as well as number of referrals made relevant to diabetes/ischaemic heart disease/hypertension care)
  - Patient Assessment of Chronic Illness Care (PACIC)/Assessment of Care for Chronic Conditions (ACC)
  - Patient self-report lifestyle risk factors
  - Referral Questionnaire (Developed for this study relating to experience of referral)

*Practice tools*:

| **Name** | **Description** | **Method** | **Relevant staff** |
| --- | --- | --- | --- |
| Pre Practice Visit Questionnaire (PPVQ) | Sent before first data collection visit. It gathers demographic information on the practice (Staff mix, patient population, etc.) | Survey (20mins) | Principal GP or Practice Manager |
| Team Climate Inventory (TCI) | Measures team environment | Questionnaire  (15mins) | All staff |
| Measure of Multidisciplinary Linkages (MoML-GP version) | Measures working relationship between GP and other health professionals | Questionnaire  (15mins) | GP only |
| Practice Profile Interview (PPI) | Examines the practice’s systems, processes, roles, and responsibilities relevant to chronic disease care. | Interview (45mins) | Principal GP or Practice Manager |
| Clinical Care Interview | Examines GP’s care provided to patients with Type 2 diabetes and ischaemic heart disease/hypertension | Interview (30mins) | GPs |

Patient tools

| **Name** | **Description** | **Method** |
| --- | --- | --- |
| Assessment of Care for Chronic Conditions/Patient Assessment of Chronic Illness Care | Assesses patient perception of the care received by GP. | Questionnaire (20mins) |
| SF-12 | Assesses health and restrictions on everyday activities | Questionnaire  (15mins) |
| Referral Questionnaire | Assesses patient experience of referral process, including satisfaction, expectation and knowledge | Questionnaire  (15mins) |

Referral service tools

| **Name** | **Description** | **Method** |
| --- | --- | --- |
| Measure of Multidisciplinary Linkages (MoML-Referral service version) | Measures working relationship between GP and other health professionals | Questionnaire  (15mins) |

***Facilitator***

*Background*

The intervention facilitators were employees (Project Officers) of the respective DGPs with a health-related background: nursing, overseas medical training or public health.

*Training session*

All facilitators received a half-day training workshop, prior to the workshop facilitators were given the practice workbook and the intervention facilitator manual which outlined the details of the intervention. The purpose of the training was to give facilitators an opportunity to run through their preparation for various practice scenarios, and also to ask questions. Scenarios that were covered:

- Organise and co-facilitate the multidisciplinary education session (hosted by the Division)
- Interacting with practices during the three structured practice visits – at the beginning, at the middle, and at the end of the 6 month intervention.
- Introducing components of effective teamwork
- Introducing Medicare items
- Introducing the Plan-Do-Study-Act cycles
- Dealing with different levels of engagement with the project within the practice
- Giving feedback to practices

The Team-link Project Manager communicated with the facilitators regularly (by emails and phone calls) to ensure fidelity to the research protocol and also organised face-to-face meetings for the facilitators to discuss any concerns and work as a team to support each other in addressing any issues arose during the intervention.

**Intervention**

1. GP Training Evening Workshop – linking together for chronic disease care

This evening session (approximately 2 - 2 ½ hours) was meant to draw together the practice staff participating in this study, together with other health professionals involved in chronic disease care. Intended health professionals include practice nurses, podiatrists, endocrinologists, cardiologists, optometrists, diabetes educators, dietitians, cardiac rehabilitation workers, exercise physiologists, Aboriginal health workers, as well as psychologists/mental health workers. In particular, the inclusion of health professionals from the public health system – in particular, diabetes clinics and cardiac rehabilitation programs is important.

Content covered in the session include:

- Optimal patient outcomes is achieved through multidisciplinary care, and a good working relationship between those involved in providing care so that seamless, continuous care may be achieved.
- Different professions can contribute to the management of patients with chronic diseases. Present case studies, and allow different professions to contribute at the evening session.
- Personal relationships and links are vital in developing good working relationships. Participants will be encouraged to sit with people they don’t know, and to form groups to work on case studies so that there is a blend of professions.
- Communication skills will also be important. Use TCAs as an opportunity to discuss communication. In particular, highlight that TCAs are meant to promote communication in sharing care.
- While TCAs can help you introduce the issue of communication, be careful how you approach this topic in the evening session. When you consider that TCAs benefit GPs financially to a greater extent than it does other professionals, it does not make sense to harp on the obvious. Save discussions about MBS items making teamwork financially viable for when you visit practices.
- Discuss referral and reply information. Present the literature highlighting dissatisfaction with content and timeliness of referral and reply information.

2. Three structured practice visits (approximately **60 – 90 minutes each**).

*Initial practice visit*

- Establish a liaison person in the practice who will be your point of contact for the duration of the project.
- Outline the intervention to all staff – focusing on working relationships and teamwork with external providers, setting up systems and processes to facilitate sharing care, and making teamwork financially viable.
- Introduce practice resources – referral directory, aids to referral (eg referral forms, cards with referral criteria etc), TCA care plan templates, billing systems
- Ask staff to reflect on potential areas for improvement in light of what they learnt at the evening session. Where are there gaps? What changes do they want to make in the practice? Encourage the staff to reflect and identify a few small goals to start. Using your own knowledge of the practice and the content in the practice workbook, you can guide them to explore problem-solving from a systems/process approach, a communication/relationships approach, etc. There is also a section at the back of the practice workbook with a suggested list of changes to implement.
- Work with differing levels of motivation to engage practice staff with the project (see Objective 6 for more detail).
- Discuss the Plan-Do-Study-Act cycle together with goal setting (SMART acronym). Encourage them to engage in setting one goal and to start to engage in the PDSA cycle.
- Encourage practices to call you if they experience any difficulties.
- Encourage practices to set additional goals and to test them out. Remember – the next time you see them will be about 3 months away!

*Second practice visit*

- - Evaluate outcome of goals set. Ask practice staff to reflect on (i) what worked, (ii) what didn’t work, (iii) what they could have done differently, and (iv) what other areas for improvement that this revealed.
  - If direct communication has occurred with other health professionals, ask what impact this has had on working relationships. Areas to explore include increased understanding of the other person’s role, increased understanding of the referral process between the practice and the health professional, better communication, etc. Because an improved working relationship is our target in this study, try hard to ask questions to capture this, and praise practice staff for achieving this.

*Third practice visit*

- At the third practice visit, help practices review all of the goals they have set and worked on throughout the past 6 months. The aim of this is to help practices reflect on the gains they have made, the pitfalls they have found, and areas for improvement.
- Ask practice staff to also reflect on their ability and comfort levels in engaging in PDSA cycles. This is the generic skill that they will be able to transfer to other projects/areas.
- Discuss sustainability of the gains that they have made. What do they need to help them keep up to date on a regular basis? How can their Division support them in this?

3. Ongoing contact with practice through face to face and phone contact (on need basis, ranged from a total of 6 – 12 times during a 6 months period)

- The purpose of this ongoing contact is for you to touch base with practices and to assist with troubleshooting. This will involve checking on what has been done since the last contact, what has worked, what hasn’t worked, what they could change in response if something didn’t work, and whether the task has exposed new areas for improvement.
- Regarding troubleshooting, be mindful of ending up in a position where practices turn to you for answers. The purpose of this intervention is to enable practices to be proactive in enhancing the process of sharing care, not to just implement ideas that someone else suggests. If the solution to their problem is obvious to you, try to steer the practice staff in the right direction with some artful questioning so that they can come up with the answer themselves.

4. Liaison with referral services where necessary

Sometimes liaising with referral services that your practices share care with may be necessary. It will require a balancing act between bringing the two parties together (whether in person or over the phone/email/fax) and you ending up being the ‘go between’. It may be necessary to bring the health professionals together in a meeting, perhaps arranging a visit to the practice.
